# Rupture of the Spleen

**Published:** 1850-01

**Authors:** M. G. Whitney

**Affiliations:** Kingston, Pennsylvania


*Rupture of the Spleen. Autopsy.* By M. G. Whitney, M. D., of Kingston, Pennsylvania.

The patient was a man named —— Young, living in Wyoming Valley, aged about 40, a coal laborer, very large and muscular, subject to intermittent fever. On the night of the 24th of November, 1849, he was engaged with a party serenading a newly married couple, when, after drinking somewhat freely, and being partially intoxicated, a difficulty arose between him and one of his companions. In a struggle which ensued, Young being nearly down to the ground, was struck by his antagonist with the clenched fist, two or three blows on the left side, over the region of the stomach and spleen. Very soon it was observed that he was severely injured. He groaned; had difficulty of breathing; his extremities became cold; the pulse ceased at the wrists, and in about fifteen minutes from the time of the scuffle he died.

*Inspectio cadaveris.*—About 38 hours after death, I made an examination. The body was altered very little by the process of decomposition. It was a little discolored on the back. The abdomen was much distended and tense; on opening it, a large quantity of fluid and coagulated blood was found in the cavity. The viscera were carefully removed, and on inspection, the spleen was found about three times the normal size, of a dark greyish color, and having three rents or ruptures on the convex side, extending transversely across its body. There was a large quantity of coagula around and under the stomach and spleen; all the other abdominal viscera had a healthy appearance. I did not measure the spleen, but should think it was about ten inches long, four or five inches broad, and about four inches thick at the thickest part. The rents were about two inches apart, and extended into its substance about one inch. No further examination was made.

